# Supplementary material for: Astrocytic TDP-43 dysregulation impairs memory by modulating antiviral pathways and interferon-inducible chemokines
Source: Sci Adv. 2023 Apr 19;9(16):eade1282. doi: 10.1126/sciadv.ade1282 (PMC10115456; doi:10.1126/sciadv.ade1282)
Supplement: Supplementary file 1 — Figs. S1 to S7 Tables S1 to S4 [file sciadv.ade1282_sm.pdf]

Science Advances
AAAS

Supplementary Materials for

Astrocytic TDP-43 dysregulation impairs memory by modulating antiviral pathways and interferon-inducible chemokines

Avital Licht-Murava *et al.*

Corresponding author: Anna G. Orr, ago2002@med.cornell.edu; Adam L. Orr, alo2012@med.cornell.edu; Avital Licht-Murava, talilicht@gmail.com

Sci. Adv. **9**, eade1282 (2023)
DOI: 10.1126/sciadv.ade1282

The PDF file includes:

Figs. S1 to S7
Tables S1 to S4

Other Supplementary Material for this manuscript includes the following:

Extended Data for the Main Figures
Extended Data for the Supplementary Figures
Supplementary Uncropped Western Blot Images

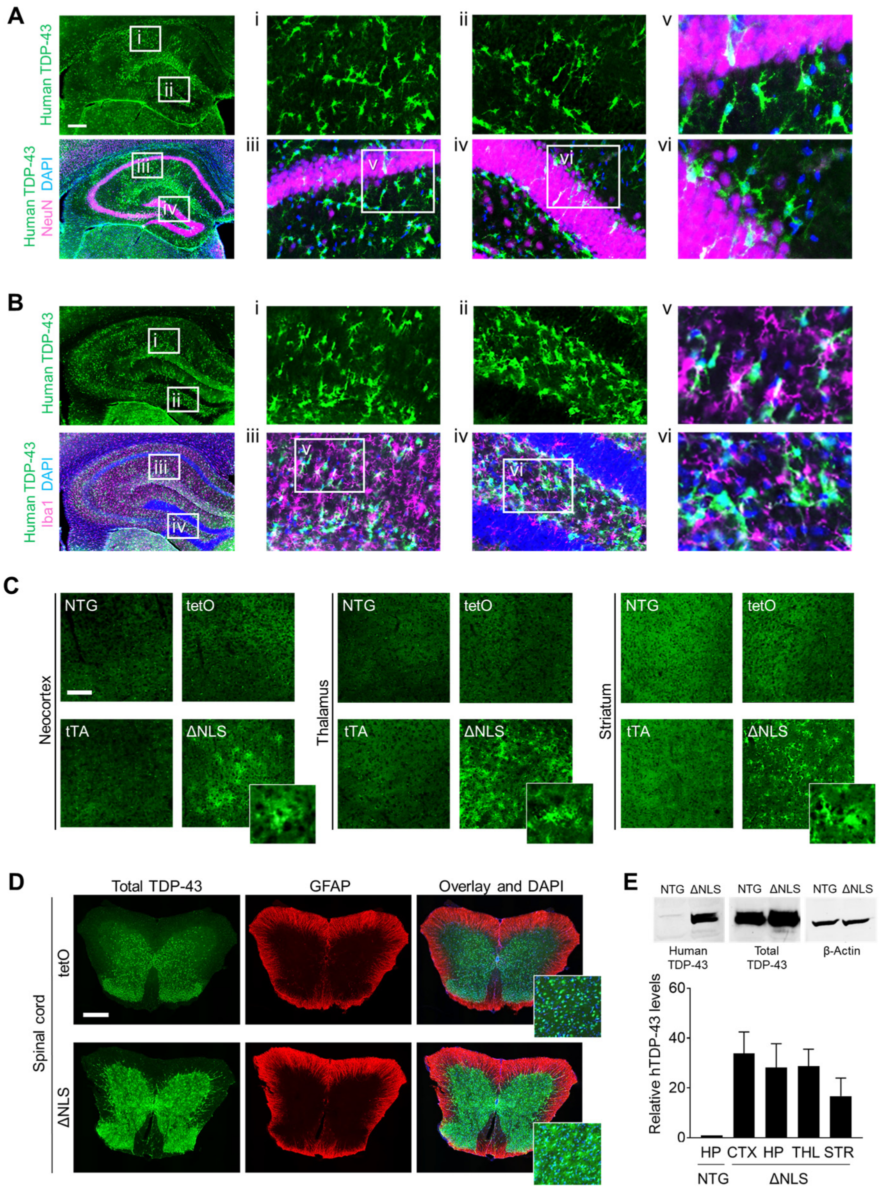

Fig. S1. Additional characterization of transgenic mice with inducible TDP-43 alterations in astrocytes. (**A**–**B**) Representative images of human TDP-43 (green) immunoreactivity in hippocampal sections from ΔNLS mice co-immunolabeled for the neuronal marker NeuN (A) or microglial and macrophage marker Iba1 (B) (magenta). DAPI (blue) was used to visualize nuclei. Insets show magnified views. Scale bar: 300 µm. n = 4–8 mice per genotype. (**C**) Representative images of human TDP-43 (green) immunoreactivity in indicated brain regions of 3-month-old nontransgenic (NTG) controls, singly transgenic *hGFAP*-tTA (tTA) and *tetO*-TDP43-ΔNLS (tetO) controls, and doubly transgenic ΔNLS mice. (**D**) Immunolabeling for total (mouse and human) TDP-43 levels (green) and the astrocyte marker GFAP (red) in the spinal cord of tetO controls and doubly transgenic ΔNLS mice. DAPI (blue) was used to visualize nuclei. Insets in (C and D) show magnified views. Scale bars: 100 µm (C), 300 µm (D). (**E**) Western blot images (top) and quantification (bottom) for human (h) and total (t) TDP-43 in different brain regions of NTG controls and ΔNLS mice. Representative images show Western blotting of hippocampal samples for indicated proteins. TDP-43 levels were normalized to β-actin levels. n = 4–5 ΔNLS mice per brain region.

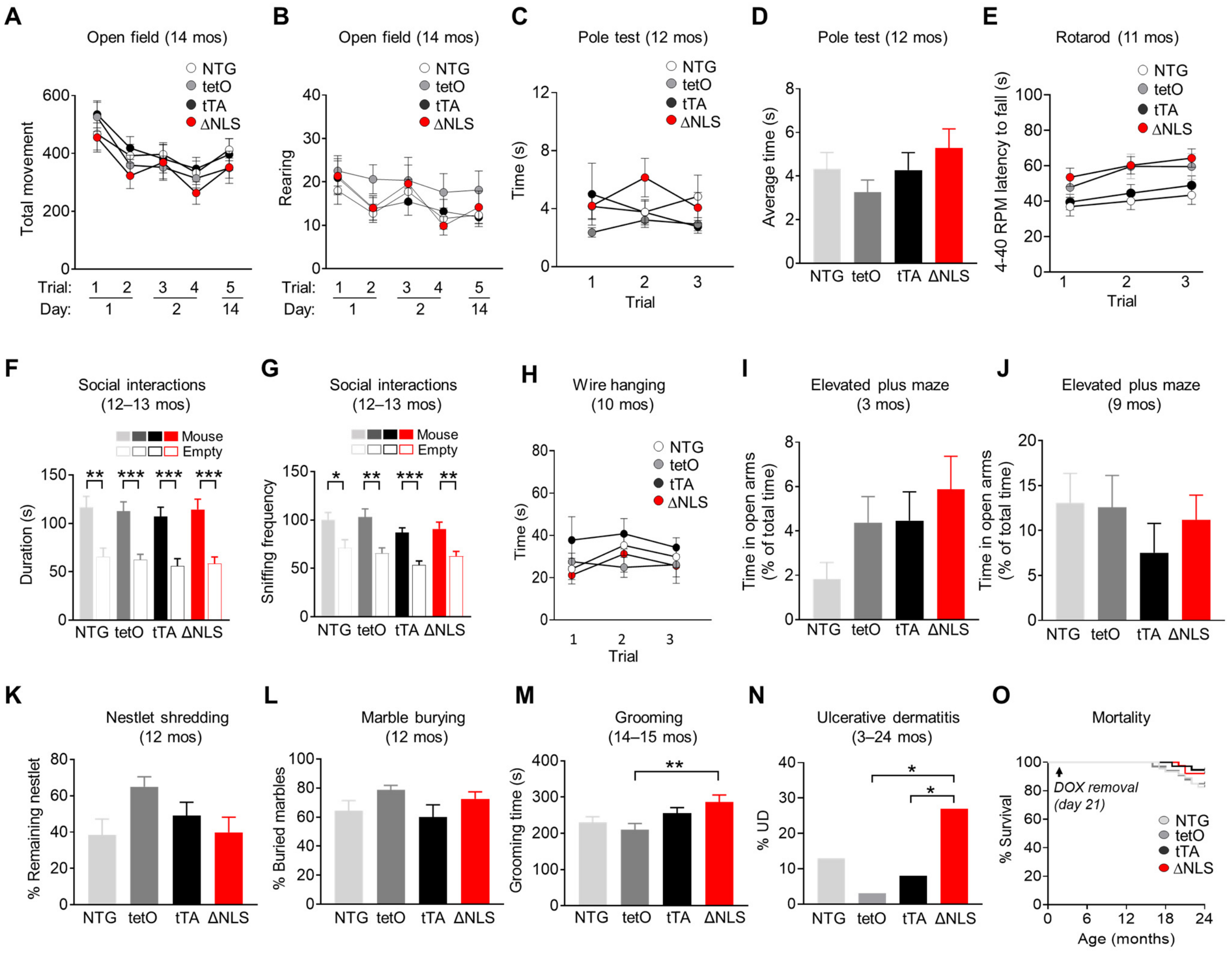

Fig. S2. Additional behavioral characterization of transgenic mice with inducible TDP-43 alterations in astrocytes. (**A**–**N**) Nontransgenic (NTG) controls, singly transgenic *hGFAP*-tTA (tTA) and *tetO*-TDP43-ΔNLS (tetO) controls, and doubly transgenic ΔNLS mice were assessed for exploratory behavior in the open field (**A**–**B**), pole test (**C**–**D**), motor behavior on the Rotarod (**E**), interactions with an unfamiliar mouse as compared to an empty wire cup (Empty) as a measure of sociability (**F**–**G**), wire hanging test (**H**), anxiety-related behavior in the elevated plus maze (**I**–**J**), nestlet shredding (**K**), and marble burying in bedding (**L**). Mice were also assessed for time spent grooming after a water mist (**M**) and incidence of ulcerative dermatitis (**N**). Ages of mice (months, mos) are indicated in each panel. (**O**) Early mortality was assessed for up to 24 months of age. (A–O) Student's t test with Welch's correction: *p < 0.05, **p < 0.01, ***p < 0.001 vs. Empty (F–G); One-way ANOVA: $F(3, 64) = 3.63$, p = 0.0175 (M); Dunnett's post-hoc test: **p < 0.01 vs. tetO (M). Fisher's exact test (two-sided): p = 0.023 (overall); *p = 0.035 vs. tTA, p = 0.007 vs. tetO, p = 0.16 vs. NTG (N). n = 15–17, 33 females and 30 males (A–B); 12–15, 32 females and 21 males (C–D); 12–15, 29 females and 26 males (E); 13–16, 30 females (F–G) and 27 (F) or 26 (G) males; 12–14, 28 females and 26 males (H); 12–17, 26 females and 30 males (I); 14–19, 34 females and 31 males (J); 12–15, 29 females and 26 males (K–L); 15–21, 36 females and 33 males (M); 35–40, 82 females and 68 males (N); 33–46, 65 females and 89 males (O) per genotype.

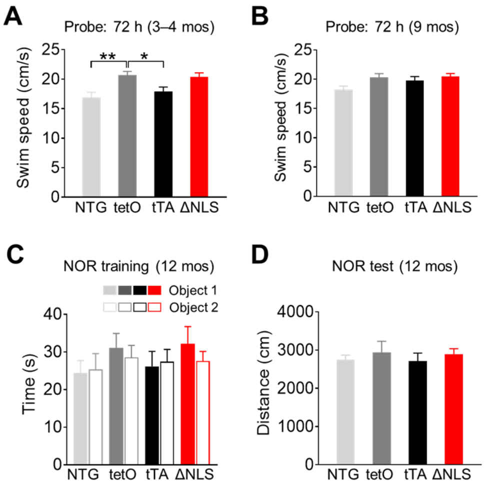

Fig. S3. Morris water maze swim speeds and novel object recognition in transgenic mice with astrocytic TDP-43 alterations. (**A**–**B**) Nontransgenic (NTG) controls, singly transgenic *hGFAP*-tTA (tTA) and *tetO*-TDP43-ΔNLS (tetO) controls, and doubly transgenic ΔNLS mice were assessed for swim speeds in probe trials at indicated time-points (hours, h) after hidden platform training. (**C**) Exploration of objects during novel object recognition (NOR) training prior to the testing day. (**D**) Total exploration during NOR testing. Ages of mice (months, mos) are indicated in each panel. One-way ANOVA: $F(3, 48) = 6.1$, p = 0.0013 (A); $F(3, 66) = 2.98$, p = 0.038 (B); $F(3, 51) = 0.90$, p = 0.448 (C); $F(3, 4) = 0.335$, p = 0.80 (D). Dunnett's post-hoc test: *$p<0.05$, **$p<0.01$ vs. tetO. n = 12–14, 27 females and 26 males (A); 15–22, 37 females and 33 males (B); 12–15, 29 females and 26 males (C); 11–13, 24 females and 24 males (D) per genotype.

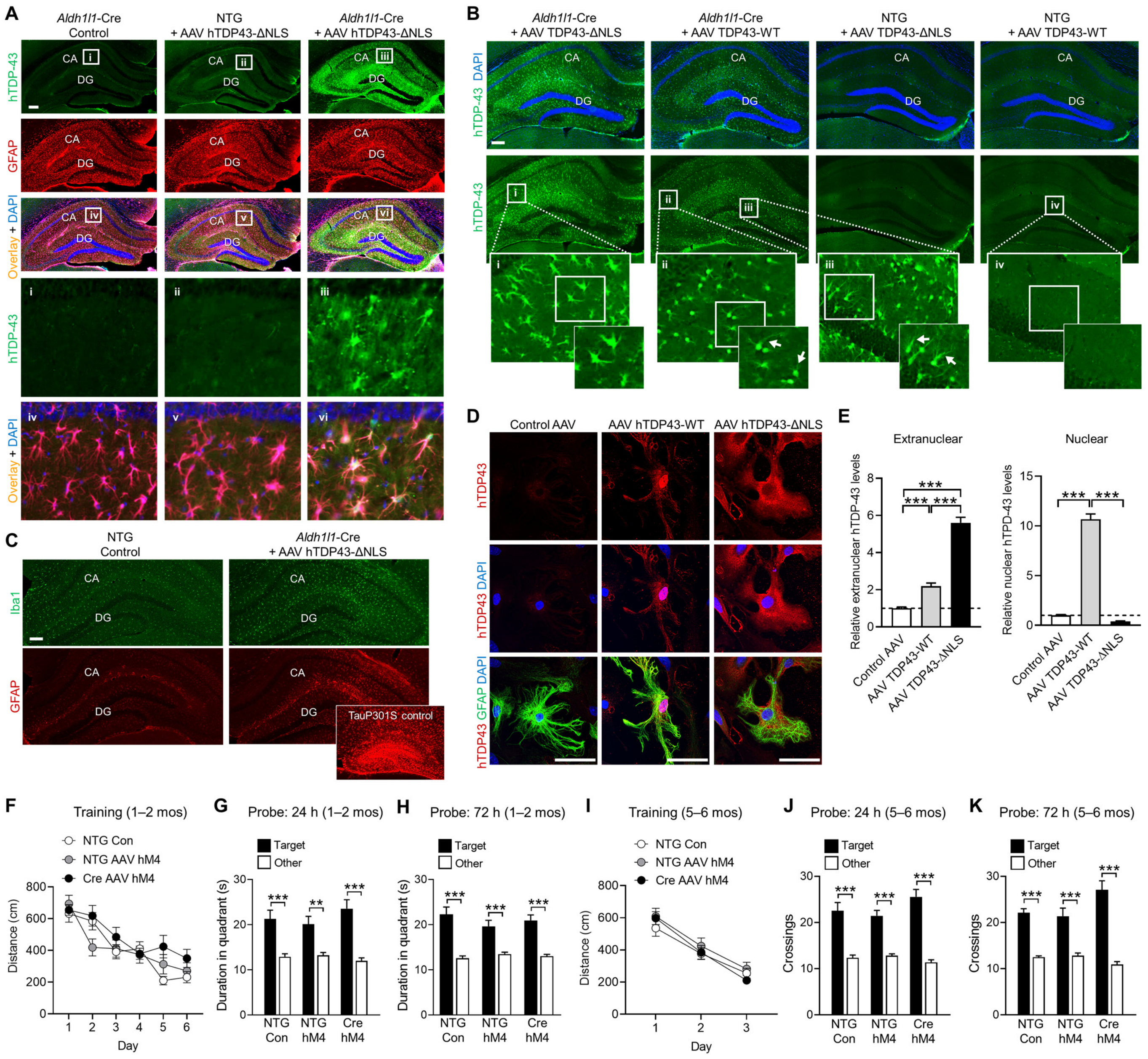

Fig. S4. Additional characterization of AAV vectors in mice and cultured astrocytes. (**A**–**C**) Representative images of hippocampal immunolabeling for human TDP-43 (green), astrocytic marker GFAP (red) and/or microglial/macrophage marker Iba1 (green) in transgenic *Aldh1l1*-Cre or littermate nontransgenic (NTG) control mice injected with AAV PHP.eB-*hGfaABC$_1$D*-DIO-hTDP43-ΔNLS, AAV PHP.eB-*hGfaABC$_1$D*-DIO-hTDP43-WT, or saline (Control). Mice were assessed 9 months (A) or 3 weeks (B) after intracranial injections. Yellow indicates overlay of green and red channels. DAPI (blue) was used to visualize nuclei. Inset in (C) shows hippocampal GFAP immunoreactivity in a transgenic TauP301S mouse as a positive control for hippocampal astrogliosis. Insets i–vi (A) and i–iv (B) show magnified views. (**D**–**E**) Representative images (D) and quantification (E) of immunolabeling for human TDP-43 in primary astrocytes derived from *Aldh1l1*-Cre mice and transduced with the indicated AAV vectors: control AAV PHP.eB (2.38E+10 Vg/well), AAV PHP.eB-*hGfaABC$_1$D*-DIO-hTDP43-WT (6.00E+10 Vg/well), or AAV PHP.eB-*hGfaABC$_1$D*-DIO-hTDP43-ΔNLS

(2.90E+11 Vg/well). Levels of hTDP-43 protein expression were similar among AAV vectors as assessed by Western blotting (data not shown). Cultures were co-immunolabeled for human TDP-43 (red) and the astrocytic marker GFAP (green). Individual astrocytes were analyzed for levels of hTDP-43 immunoreactivity in different subcellular regions as indicated. One-way ANOVA: $F(2, 101) = 144.9$, $p<0.0001$ (extranuclear); $F(2, 101) = 424.2$, $p<0.0001$ (nuclear). Bonferroni's post-hoc test: ***p<0.001 vs. indicated group. n = 30–37 cells from 3 culture wells per condition. Scale bars: 200 µm (A–C), 50 µm (D). (**F**–**K**) Nontransgenic (NTG) or transgenic *Aldh1l1*-Cre (Cre) littermates were injected with AAV PHP.eB-*hGfaABC$_1$D*-DIO-hM4Di-mCherry (hM4) or saline (Con) at 5–6 months of age and tested in the Morris water maze at 1–2 or 5–6 months after AAV injections, as indicated. (**F**, **I**) Distance traveled to reach the platform during hidden platform training (four trials per session, one session per day). Repeated measures two-way ANOVA: $F(2, 50) = 1.96$, $p = 0.15$ for group effect (F); $F(2, 47) = 0.96$, $p = 0.39$ for group effect (I). n = 17 NTG/Con, 18 NTG/hM4, and 18 Cre/hM4 mice (27 females, 26 males) (F); n = 14 NTG/Con, 18 NTG/hM4, and 18 Cre/hM4 mice (25 females, 25 males) (I). (**G–H, J–K**) Probe trials were conducted 24 or 72 h after completion of training, as indicated. Durations in target and non-target (Other) quadrants. Student's *t* test with Welch's correction: **p < 0.01, ***p < 0.001 vs. Other. n = 15 NTG/Con, 19 NTG/hM4, and 18 Cre/hM4 mice (G–H); n = 14 NTG/Con, 18 NTG/hM4, and 18 Cre/hM4 mice (J–K).

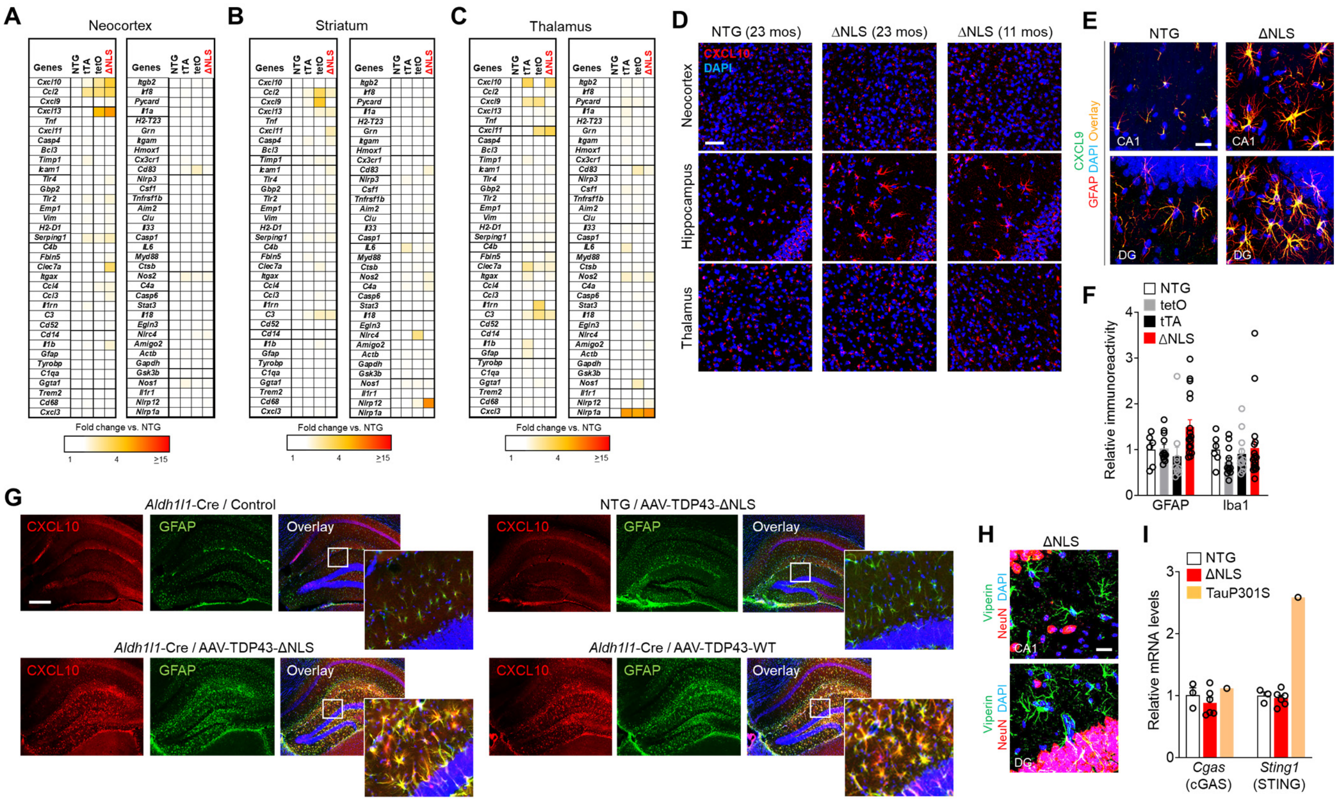

Fig. S5. Additional characterization of gene expression in transgenic mice with astrocytic TDP-43 alterations. (**A**–**C**) Relative RNA levels in indicated brain regions of 11-month-old littermate nontransgenic controls (NTG), singly transgenic *hGFAP*-tTA (tTA) and *tetO*-TDP43-ΔNLS (tetO) controls, and doubly transgenic TDP-43-ΔNLS mice (ΔNLS). (**D**) Representative images of CXCL10 immunoreactivity (red) in indicated brain regions of NTG and ΔNLS mice at different ages. DAPI (blue) was used to visualize nuclei. (**E**) Representative images of CXCL9 immunoreactivity (green) in the CA1 region or dentate gyrus molecular layer (DG) of 11-month-old NTG controls and doubly transgenic ΔNLS mice. Sections were co-immunolabeled for the astrocyte marker GFAP. Yellow indicates overlay of green and red channels. DAPI (blue) was used to visualize nuclei. (**F**) Quantification of relative GFAP and Iba1 immunoreactivity (integrated brightness) in the hippocampal formation of 11-month-old NTG, tetO, tTA, and ΔNLS mice. One-way ANOVA: $F(3, 42) = 3.6$, $p = 0.021$ (GFAP); $F(3, 44) = 0.74$, $p = 0.53$ (Iba1). n = 6–18 regions of interest (ROIs) from 3–9 mice per genotype. (**G**) Representative images of CXCL10 (red) and GFAP (green) immunoreactivity in hippocampal sections from 13-month-old nontransgenic (NTG), transgenic *Aldh1l1*-Cre (Cre), or littermate nontransgenic (NTG) control mice injected with AAV PHP.eB-*hGfaABC$_1$D*-DIO-hTDP43-ΔNLS, AAV PHP.eB-*hGfaABC$_1$D*-DIO-hTDP43-WT, or saline (Control). Mice were assessed 9 months after intracranial injections. DAPI (blue) was used to visualize cell nuclei. Insets show magnified views of boxed regions. (**H**) Representative images of viperin immunoreactivity (green) in the CA1 region or dentate gyrus molecular layer (DG) of 11-month-old NTG controls and doubly transgenic ΔNLS mice. Sections were co-immunolabeled for the neuronal marker NeuN (red). Viperin did not overlap with NeuN. DAPI (blue) was used to visualize nuclei. (**I**) Hippocampal *Cgas* and *Sting1* RNA levels in 11-month-old NTG controls and ΔNLS mice, and a transgenic TauP301S mouse as a positive control. n = 3–6 mice per genotype; n = 1 for TauP301S. Scale bars: 50 µm (D), 20 µm (E, H), 400 µm (G).

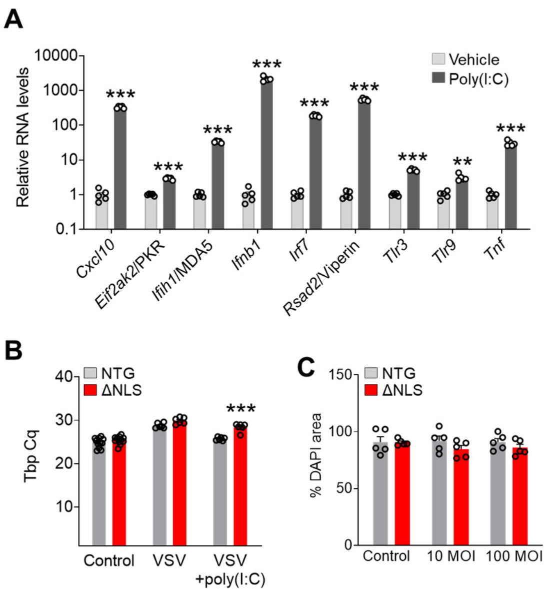

Fig. S6. Additional characterization of antiviral responses in isolated astrocytes. (**A**) Primary astrocytes (DIV 9) derived from nontransgenic mice were acutely transfected with polyinosinic-polycytidylic acid (poly(I:C)) (0.66 µg/µl in lipofectamine 3000) or treated with vehicle and were collected after 24 h for RNA analyses. RNA levels were normalized to TATA-binding protein (*Tbp*) and expressed as fold change relative to vehicle-treated controls. Student's *t* test with Welch's correction: **$p < 0.01$, ***$p < 0.001$ vs. Vehicle. n = 5 wells per treatment condition. (**B**–**C**) Primary astrocytes (DIV 9) derived from NTG or doubly transgenic ΔNLS mice were infected with vesicular stomatitis virus (VSV, 100 MOI) (B) or adenovirus tagged with eGFP at indicated MOIs (C). Some wells were also transfected with poly(I:C) as a control 5 h before viral infection. To compare cell density across treatments and genotypes, RNA levels of the control gene *Tbp* (B) or relative DAPI fluorescence (C) were assessed 24 h after infection. Two-way ANOVA: $F(2, 42) = 5.63$, $p = 0.007$ for interaction effect (B); $F(2, 24) = 0.54$, $p = 0.591$ for interaction effect (C). Bonferroni post-hoc test: ***$p < 0.001$ vs. NTG. n = 6–12 (B) and 5 (C) culture wells per genotype and treatment condition.

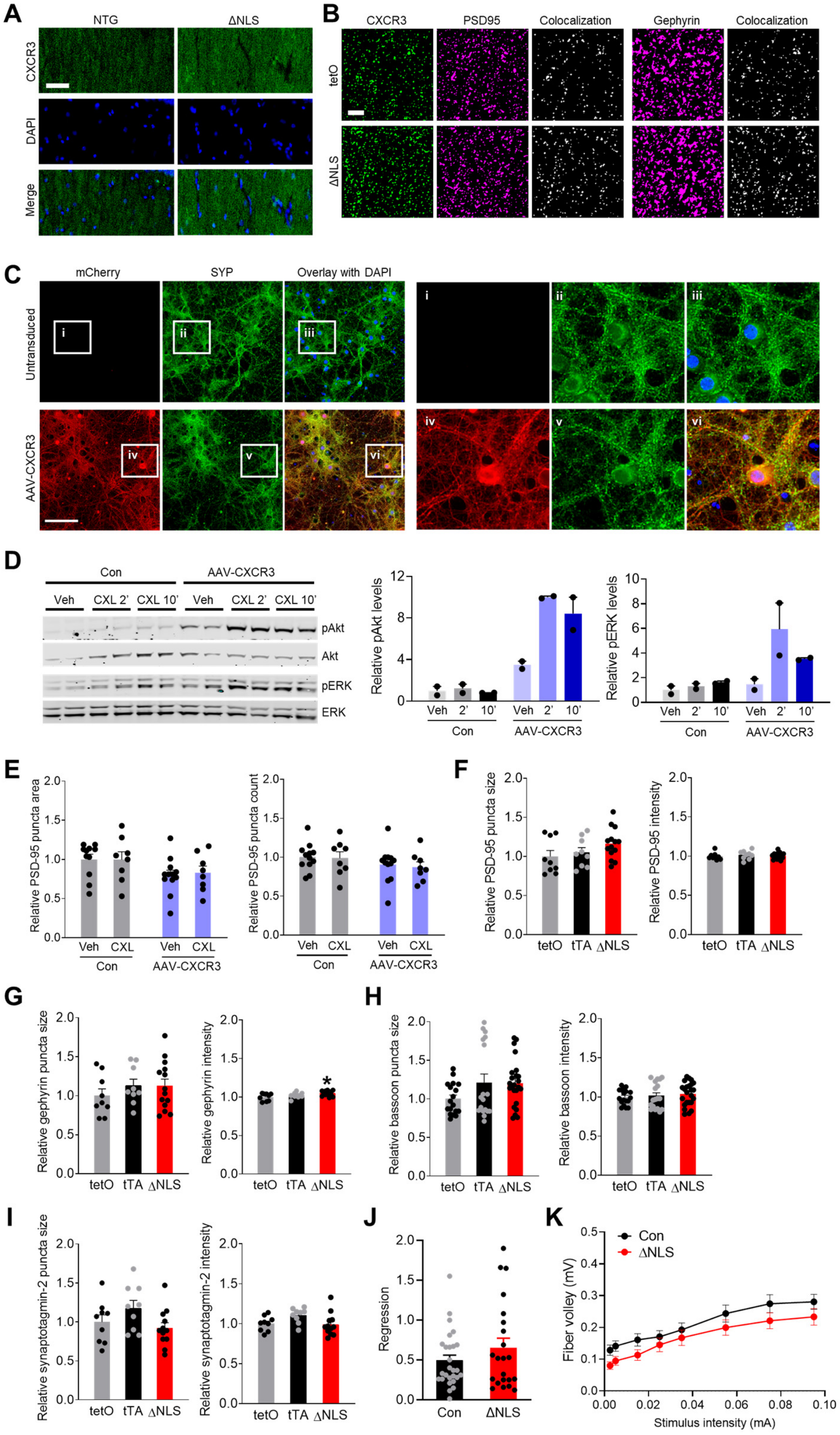

Fig. S7. Characterization of CXCR3 expression, AAV-mediated transduction in isolated neurons, synaptic markers, and neuronal activity. (**A**) Representative images of hippocampal immunoreactivity for CXCR3 in the CA1 radiatum parenchyma in NTG and ΔNLS mice. Scale bar: 50 µm. (**B**) Representative images of hippocampal immunoreactivity for CXCR3 and the synaptic marker PSD-95 or gephyrin in the CA1 region of singly transgenic

controls and doubly transgenic ΔNLS mice. Mander's overlap coefficient was used to assess colocalization. Scale bar: 5 µm. (**C**) Representative images of mCherry (red) and synaptophysin (SYP, green) immunoreactivity in primary wild-type neurons 5 days after transduction with AAV DJ-*hSyn1*-mCherry-T2A-Cxcr3-2HA-neurexin1α (AAV Syn-CXCR3). DAPI (blue) was used to visualize cell nuclei. Yellow indicates overlap of green and red channels. Insets i–vi show magnified views. Scale bar: 100 µm. (**D**) Western blot images and quantification of phosphorylated and total Akt and ERK1/2 levels in Neuro2a cells untransfected (Con) or transfected with *hSyn1*-CXCR3 and then treated for 2 or 10 min with CXCL11 (CXL, 200 nM) or vehicle (Veh). n = 2 wells per treatment condition. (**E**) Quantification of PSD-95 immunoreactivity in primary wild-type neurons 5 days after transduction with AAV Syn-CXCR3 and treatment with CXCL11 (CLX, 200 nM) or vehicle (Veh) for 3 days. Control cultures (Con) were not transduced. Two-way ANOVA: $F(1, 35) = 0.055$, $p = 0.816$ for interaction effect (size); $F(1, 36) = 0.027$, $p = 0.87$ for interaction effect (count). n = 8–12 wells per condition. (**F**–**I**) Quantification of hippocampal immunoreactivity for synaptic markers PSD-95 (F), gephyrin (G), bassoon (H), and synaptotagmin-2 (I) in the CA1 region of singly transgenic controls (tetO and tTA mice) and ΔNLS mice. One-way ANOVA: $F(2, 29) = 1.86$, $p = 0.17$ (size); $F(2, 29) = 0.23$, $p = 0.78$ (intensity) (F); $F(2, 29) = 0.69$, $p = 0.52$ (size); $F(2, 29) = 4.38$, $p = 0.02$ (intensity) (G); $F(2, 57) = 2.33$, $p = 0.11$ (size); $F(2, 57) = 0.38$, $p = 0.68$ (intensity) (H); $F(2, 27) = 2.37$, $p = 0.11$ (size); $F(2, 27) = 2.94$, $p = 0.07$ (intensity) (I); Dunnett's post-hoc test: *$p < 0.05$ vs. tetO. n = 9–24 per genotype (F–I). (**J**–**K**) Hippocampal slice recordings in control and ΔNLS mice. (J) Linear regression between fEPSP slopes and fiber volley amplitudes. (K) Fiber volley amplitudes measured at increasing stimulus intensities as indicated. Mixed-effects model: $F(1, 51) = 2.61$, $p = 0.11$ for genotype effect. n = 22–28 recordings per genotype.

Table S1. Clinicopathological information for human brain tissue analyses shown in Fig. 1.

**Cases (n)**	**PMI (hours)**	**M/F Ratio**	**Age (years)**	**Individual NIA-Reagan Scores**
Con (9)	4.8 +/- 2.2	2:1	80.2 +/- 7.3	4, 4, 4, 4, 1, 1, 1, 0, NA
AD (10)	6.0 +/- 5.3	4:1	75.6 +/- 10.4	3, 3, 3, 3, 3, 3, 3, 3, 3, 3
s-FTD (9)	6.4 +/- 4.8	1:2	75.8 +/- 13.2	4, 4, 1, 1, 1, 0, NA, NA, NA
f-FTD (4)	17.8 +/- 12.6	3:1	68.5 +/- 3.9	NA, NA, NA, NA

Values indicate mean +/- SD; Postmortem interval (PMI); Male-to-female ratio (M/F); Folstein Mini Mental State Examination (MMSE); NIA Reagan criteria for the postmortem diagnosis of Alzheimer's disease were developed jointly by the National Institute on Aging and the Reagan Institute*. These criteria suggested that neuritic plaque density and Braak neurofibrillary staging offer probabilistic estimates of their likelihood for being responsible for dementia. Score 0 = not AD; 1 = low likelihood; 2 = intermediate likelihood; 3 = high likelihood; 4 = subject was not demented. NA: not available.

*Consensus recommendations for the postmortem diagnosis of Alzheimer's disease. The National Institute on Aging, and Reagan Institute Working Group on Diagnostic Criteria for the Neuropathological Assessment of Alzheimer's Disease. Neurobiol Aging 18, S1-2 (1997).

Table S2. Number of individual cells analyzed per case for human brain tissue analyses shown in Fig. 1.

**Con (case #)**	**Cells (n)**	**s-AD (case #)**	**Cells (n)**	**s-FTD (case #)**	**Cells (n)**	**f-FTD (case #)**	**Cells (n)**
1	41	1	26	1	21	1 (C9)	24
2	24	2	16	2	28	2 (C9)	21
3	36	3	18	3	27	3 (GRN)	22
4	43	4	18	4	28	4 (GRN)	18
5	16	5	12	5	28		
6	18	6	73	6	68		
7	18	7	20	7	16		
8	23	8	24	8	24		
9	17	9	18	9	21		
		10	24				
Total:	236		249		261		85

Table S3. Primer sequences.

**Gene**	**Forward Primer**	**Reverse Primer**
Actb	CCCTAAGGCCAACCGTGAAA	AGCCTGGATGGCTACGTACA
Aim2	TGGGCTGTTTAAAGTCCAGAA	CACCTCCATTGTCCCTGTT
Amigo2	GCAGAGAGCTGCTGTGTC	GTGGGGCACATTCCTGAG
Bcl3	GTGGAGAACAACAGCCTGAAC	CATCTGAGCGTTCACGTTGG
C1qa	ATGGGGCTCCAGGAAATCC	TCCCCTGGGTCTCCTTTAAAAC
C3	ACCTTACCTCGGCAAGTTTCT	TTGTAGAGCTGCTGGTCAGG
C4a	CAGCACCTTTGTCAAGGTTACA	GACAAAGCTCCTTCAGAGCC
C4b	TCTCACAAACCCCTCGACAT	AGCATCCTGGAACACCTGAA
Casp1	GGAGGACATCCTTCATCCTCA	GCAAAACTTGAGGGTCCCA
Casp4	CCAGACATTCTTCAGTGTGGA	CTGGTTCCTCCATTTCCAGA
Casp6	GCTCAAAATTCACGAGGTGTC	TCGTATGCGTAAACGTGGTT
Ccl2	GCCTGCTGTTCACAGTTGC	CAGGTGAGTGGGGCGTTA
Ccl3	AGATTCCACGCCAATTCATCG	GCCGGTTTCTCTTAGTCAGGA
Ccl4	GCCCTCTCTCTCCTCTTGC	GAGAAACAGCAGGAAGTGGGA
Cd14	AGCCTTTCTCGGAGCCTATC	AGCAACAAGCCAAGCACAC
Cd52	GCCCAGGAAGATTTCAGGAT	CCCAAGGATCCTGTTTGTATCT
Cd68	CTGTTCACCTTGACCTGCT	TCACGGTTGCAAGAGAAACA
Cd83	TCCAGCTCCTGTTTCTAGGC	GGACACTGCATAGGAGAGCT
Cgas	TGGCAGCTACTATGAACATGTGA	CCTGGGGACTTCCAGTTTAAAC
Clec7a	AGAGTGAAGGGCCATGGTT	CCTGGGGAGCTGTATTTCTGA
Clu	AGCTGGCTAACCTCACAC	GACCTCTGAGTCAGAGGAATG
Csf1	CCTAGTCTTGCTGACTGTTGG	CCAATGTCTGAGGGTCTCG
Ctsb	AAGCTGTGTGGCACTGTCC	GATCTATGTCCTCACCGAACGC
Cx3cr1	GGCCTAGAGCTCAAAGAAATCC	CACAGACCTTCGATCCCAGT
Cxcl10	GGGAAGCTTGAAATCATCCCT	TCAGACATCTCTGCTCATCATTC
Cxcl11	TGCTGAGATGAACAGGAAGG	CGCCCCTGTTTGAACATAAG
Cxcl13	TGAGGCTCAGCACAGCAA	ATGGGCTTCCAGAATACCGTG
Cxcl3	GCCACACTCCAGCCTAGC	GCCACAACAGCCCCTGTA
Cxcl9	CCATGAAGTCCGCTGTTCTT	GCATTCCTTATCACTAGGGTTCC
Cxcr3	ACAAGTGCCAAAGGCAGAG	GGCATCTAGCACTTGACGTTC
Ddx58	GCAGAACTGGAACAGGTCG	GTTCGAAGTCCGGGATGC
Egln3	CCGGCTGGGCAAATACTATG	CCACATGGCGAACATAACCTG
Eif2ak2	GTTGTTGGGAGGGAGTTGAC	AGAGGCACCGGGTTTTGTA
Emp1	TTCGTGTTCCAGCTCTTCAC	CTGCTGGAGTTGAAGTTCCC
Fbln5	AGGAAGATGGCATTCACTGCAG	GGCTGGTTCACACACTCGT
Gapdh	CAAGGTCATCCCAGAGCTGAA	CAGATCCACGACGGACACA
Gbp2	GTAGACCAAAAGTTCCAGACAG	GATAAAGGCATCTCGCTTGG
Gfap	AGAACAACCTGGCTGCGTATA	CAGCGATTCAACCTTTCTCTCC
Ggta1	AGATCGCATTGAAGAGCCTCA	ACGGGGTCACTGTCAAAACA
Grin1	GATGGCAAGTTTGGCACACA	AGCAGCTCTCCCATCATTCC
Grin2a	AGGAGGAGTTTGTGGACCAA	CAAATCGGAAAGGCGGAGAA
Grin2b	TGCATCCGAAGCTGGTGATA	TGCAGGGACTTGTCTTTCCA
Grin2c	CGTAGACAGAGCAACCACAC	CGATGCAGAAGCCCTTACAA
Grn	GGGAAATCCTGCTTCCAGATG	TGGCAGAGTCAGGACATTCA

Gsk3b	CCCTCAAATCAAGGCACATCC	AGGTGTGTACTCCAGCAGAC
H2-D1	AGGAACCTGCTCGGCTACTA	CCCAAGTCACAGCCAGACA
H2-T23	ACGGCTGGGAAATGAGACA	GCACCTCAGGGTGACTTCA
Hmox1	CCTCACAGATGGCGTCACTT	GCTGATCTGGGGTTTCCCTC
Icam1	TTGGAGCTAGCGGACCAG	GGACCGGAGCTGAAAAGTTG
Ifih1	CTTGTCACGAACGAGATAGCC	CCAGGACATACGTGCTTTCAT
Ifnb1	CGGACTTCAAGATCCCTATGG	ACCCAGTGCTGGAGAAATTG
Ifng	ACGGCACAGTCATTGAAAGC	TGTCACCATCCTTTTGCCAG
Il18	ACAGCCTGTGTTCGAGGAT	TCACAGCCAGTCCTCTTACTT
Il1a	GGTTAAATGACCTGCAACAGGA	GAGCGCTCACGAACAGTT
Il1b	GCCACCTTTTGACAGTGATGAG	ACAGCCCAGGTCAAAGGTT
Il1r1	TGGAAGGGATGACTATGTTGG	TGAAGCCTCCCATATCTCTCA
Il1rn	TGTGCCAAGTCTGGAGATG	GCGCTTGTCTTCTTCTTTGTTC
Il33	GGTGAACATGAGTCCCATCA	CGTCACCCCTTTGAAGCT
IL6	CGATGATGCACTTGCAGAAA	ACTCCAGAAGACCAGAGGAA
Irf7	CTTCAGCACTTTCTTCCGAGA	TGTAGTGTGGTGACCCTTG
Irf8	GAGCCAGATCCTCCCTGAC	GGCATATCCGGTCACCAGT
Itgam	GACTCTCATGCCTCCTTTGG	GTGGGTCCTGGACATGTTG
Itgax	TGGCTGTAGATGACCAAACGT	TGTGGTCAGCTCCACAGTT
Itgb2	CATCCATGTGGAGGACAGTCT	CCAATCAGTACGACACCTACCA
Myd88	GCCTTGTTAGACCGTGAGGA	CCTGGTTCTGCTGCTTACCT
Nlrc4	GGCCTGCAACCTCTTTCTTA	CAGGTCTTCTTCTGTGACCTG
Nlrp12	CACCAGACCTGCAGACTC	CATGCTTTGGAGGTGAGTCC
Nlrp1a	CCCGCTATATCGTGTCTTCC	CGGTAGCACAGCTCTAGTTC
Nlrp3	TTCCCAGACACTCATGTTGC	AGAAGAGACCACGGCAGAA
Nos1	GTCAAGTACGCCACCAACAA	GGAAGTCATGCTTGCCATCA
Nos2	CTTTGCCACGGACGAGAC	TCATTGTACTCTGAGGGCTGAC
Pycard	GGAGCTCACAATGACTGTGCT	CTGCCACAGCTCCAGACTC
Rsad2	CGAGGACTGCTTCTGCTCA	CCAAGTATTCACCCCTGTCCT
Serping1	TGGCCCAATTCGATGACCATA	ACGGGTACCACGATCACAAA
Stat3	CCCCGTACCTGAAGACCAA	ACACTCCGAGGTCAGATCCA
Sting1	CCAACAGCGTCTACGAGATTC	ACATGGCAAACAGGGTCTG
Tbp	CCTTGTACCCTTCACCAATGAC	ACAGCCAAGATTCACGGTAGA
Timp1	ATGCCCACAAGTCCCAGAAC	TGCAGGCACTGATGTGCAAA
Tlr2	GGGCTTCACTTCTCTGCTT	AGCATCCTCTGAGATTTGACG
Tlr3	GATACAGGGATTGCACCCATAA	TCCCCCAAAGGAGTACATTAGA
Tlr4	GGACTCTGATCATGGCACTG	CTGATCCATGCATTGGTAGGT
Tlr9	GGAGAATCCTCCATCTCCCAA	AGAGTCTCAGCCAGCACT
Tnf	GGGTGATCGGTCCCCAAA	TGAGGGTCTGGGCCATAGAA
Tnfrsf1b	GAAGGCTCAGATGTGCTGT	CCGAGGTCTTGTTGCAGAA
Trem2	TGGGACCTCTCCACCAGTT	TGGTGTTGAGGGCTTGGG
Tyrobp	TGGTGTTGACTCTGCTGATTG	GTCTCAGCAATGTGTTGTTTCC
Vim	GATTTCTCTGCCTCTGCCAAC	CAACCAGAGGAAGTGACTCCA
VSV	TGATACAGTACA ATTATTTGGGAC	GAGACTTTCTGTTACGGGATCTGG

Table S4. Numbers of replicates shown in the main figures.

Main figure	Panel(s)	Replicates per group or condition (n)
1	B–D	9 Con, 10 s-AD, 9 s-FTD, and 4 f-FTD cases
2	B–C	9 mice for hTDP-43/GFAP in the DG, 8 mice for hTDP-43/GFAP in the CA1, and 3 mice for hTDP-43/NeuN
2	D	4–8 mice per genotype
2	E	14 NTG, 12 tetO, 15 tTA, and 14 ΔNLS mice (29 females, 26 males)
2	F–G	14 NTG, 12 tetO, 13 tTA, and 14 ΔNLS mice (27 females, 26 males)
2	I	22 NTG, 15 tetO, 16 tTA, and 17 ΔNLS mice (37 females, 33 males)
2	J–K	22 NTG, 15 tetO, 16 tTA, and 17 ΔNLS mice (37 females, 33 males)
2	M–N	22 NTG, 15 tetO, 16 tTA, and 17 ΔNLS mice (37 females, 33 males)
2	O	11 NTG, 11 tetO, 15 tTA, and 13 ΔNLS mice (25 females, 25 males)
3	C–D	6 AAV hTDP43-ΔNLS and 12 AAV hTDP43-WT-injected *Aldh1l1* -Cre mice
3	E–F	16 Cre/Con, 19 NTG/ΔNLS, and 24 Cre/ΔNLS mice (27 females, 32 males)
3	G	14 Cre/Con, 18 NTG/ΔNLS, and 19 Cre/ΔNLS mice (22 females, 29 males)
3	H	14 Cre/Con, 17 NTG/ΔNLS, and 18 Cre/ΔNLS mice (22 females, 27 males)
3	J	14 Cre/Con, 18 NTG/ΔNLS, and 19 Cre/ΔNLS mice (22 females, 29 males)
3	K	16 Cre/Con, 19 NTG/TDP-WT, and 22 Cre/TDP-WT mice (22 females, 35 males)
3	L	15 Cre/Con, 19 NTG/TDP-WT, and 21 Cre/TDP-WT mice (22 females, 33 males)
3	M–N	12 Cre/Con, 18 NTG/TDP-WT, and 17 Cre/TDP-WT mice (19 females, 28 males)
3	P	12 Cre/Con, 18 NTG/TDP-WT, and 15 Cre/TDP-WT mice (18 females, 27 males)
4	A	3–6 mice per genotype
4	B–C	3–6 mice per genotype and brain region
4	D–E, H	$\geq$ 3 mice per genotype
4	F–G	3–6 mice per genotype
5	A–B	6 culture wells per genotype
5	C–D	5–12 wells per genotype
5	E–F	4–7 culture wells per genotype from 1–2 independent cultures
5	G	8–11 culture wells per genotype from two independent cultures
5	H–I	4–5 wells per genotype
5	J–K	4–11 wells per genotype and treatment condition from 1–2 independent cultures
5	L–M	4–5 wells per genotype and treatment condition
5	N	3–10 wells per genotype and treatment condition
5	O	3–5 wells per genotype and treatment condition
6	A	6–12 wells per genotype and treatment condition
6	B	5 wells per genotype and treatment condition
6	C	3 wells per genotype and treatment condition
6	E	3–9 wells per genotype and treatment condition
7	A	3–5 mice per genotype and brain region
7	B–D	3 mice per genotype
7	E–F	8–11 mice per genotype
7	G–H	9–13 mice per genotype
8	A–B	11–14 culture wells per genotype and treatment condition
8	C	8–12 culture wells per genotype and treatment condition
8	E–F	9–11 mice per genotype
8	G	28 recordings from 6 Con male mice and 24 recordings from 5 ΔNLS male mice
8	H	36 recordings from 6 Con male mice and 24 recordings from 5 ΔNLS male mice
8	I–J	5–6 mice per genotype across 7 stimulus intervals
8	K–L	5–13 culture wells per genotype and treatment condition
9	C	20 Con / *Cxcr3*- WT, 19 ΔNLS / *Cxcr3* -WT, 17 Con / *Cxcr3* -KO, and 18 ΔNLS / *Cxcr3* -KO males
9	D–E, G	20 Con / *Cxcr3*- WT, 19 ΔNLS / *Cxcr3* -WT, 17 Con / *Cxcr3* -KO, and 17 ΔNLS / *Cxcr3* -KO males
