# Supplementary figures and images for: Astrocytic TDP-43 dysregulation impairs memory by modulating antiviral pathways and interferon-inducible chemokines
Source: Sci Adv. 2023 Apr 19;9(16):eade1282. doi: 10.1126/sciadv.ade1282 (PMC10115456; doi:10.1126/sciadv.ade1282)

Supplementary Raw Data

Fig. 5A

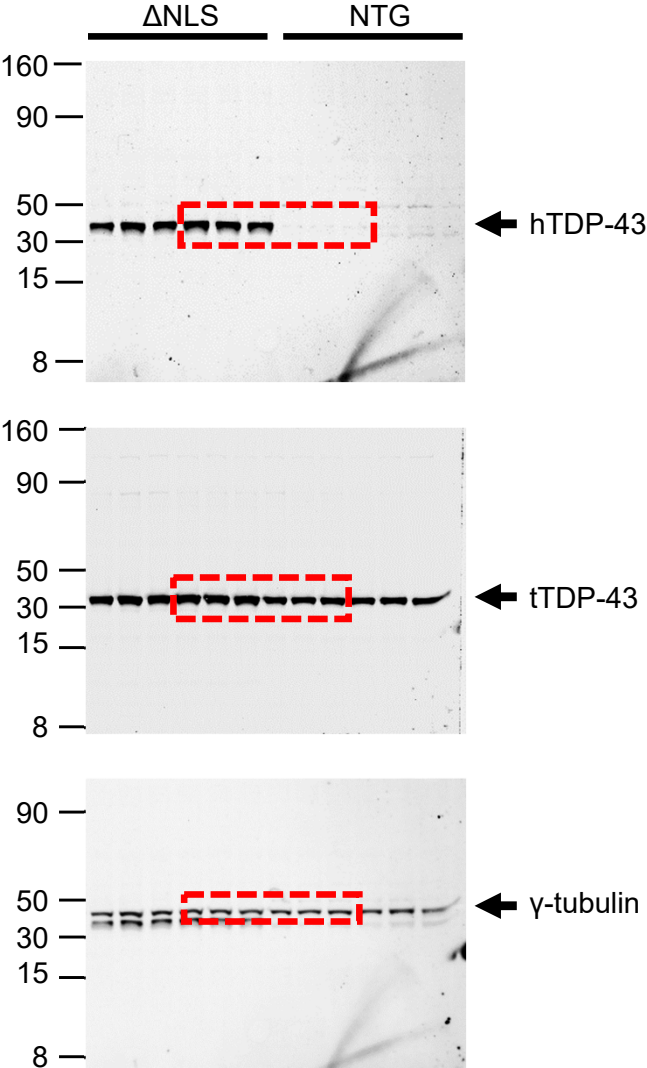

Fig. 5H

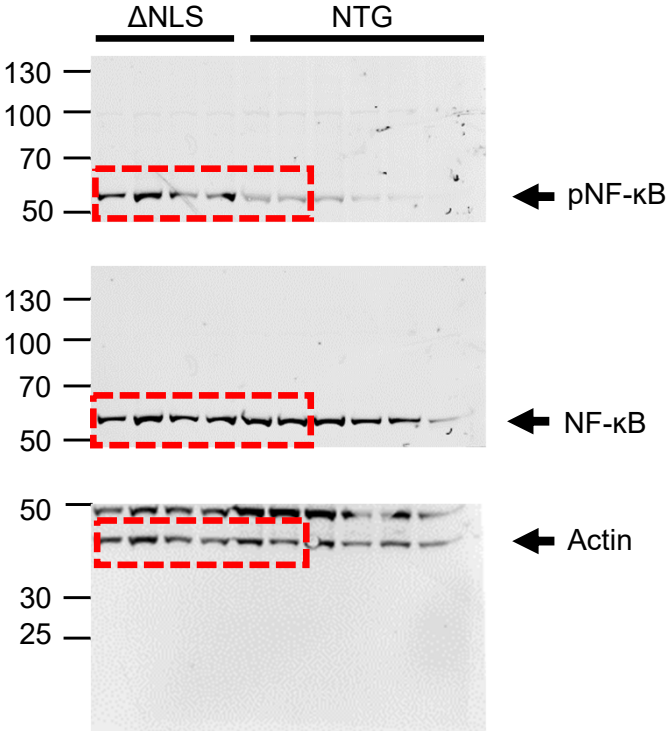

**Fig. 5L-M**

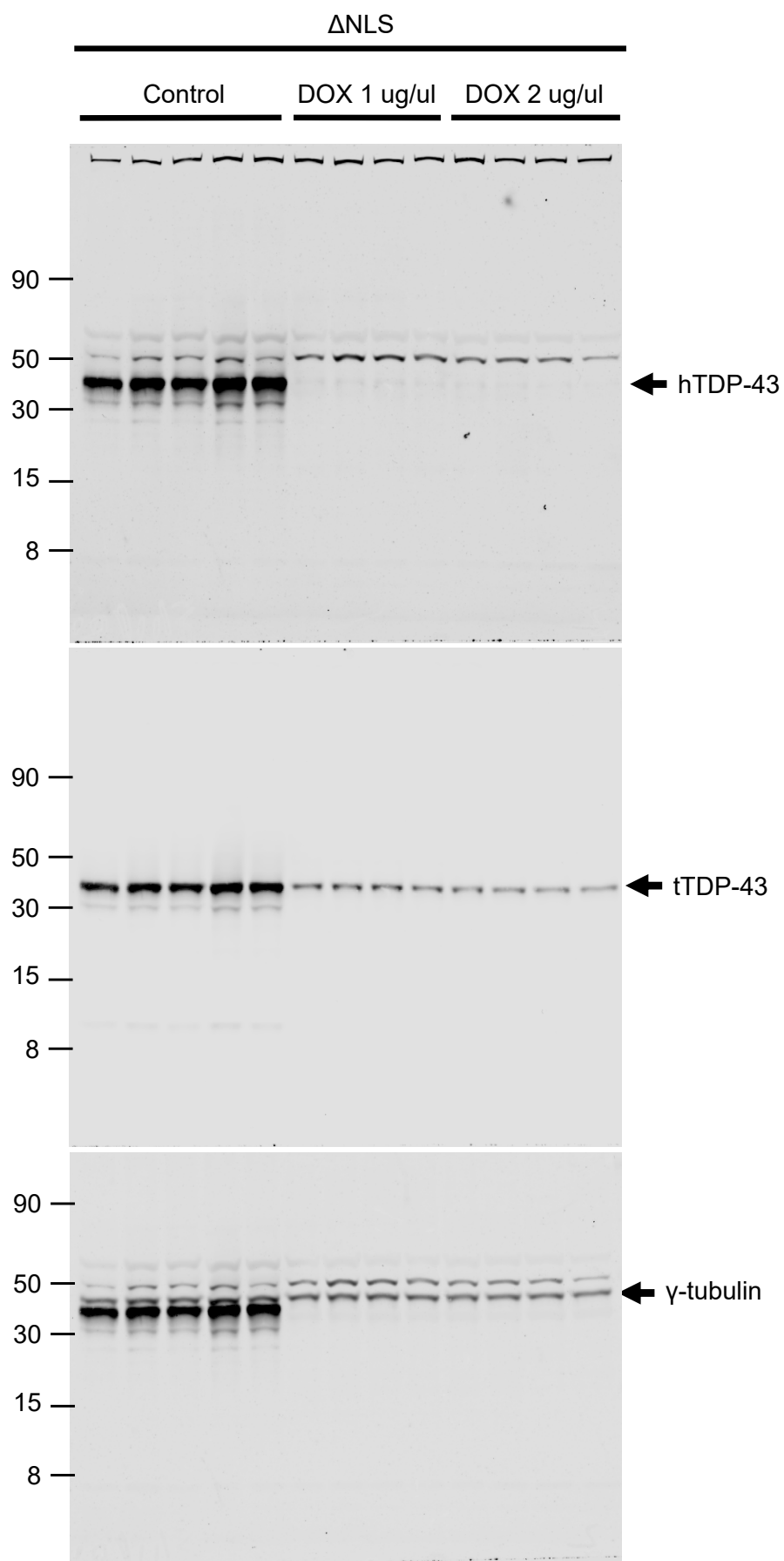

**Fig. 50**

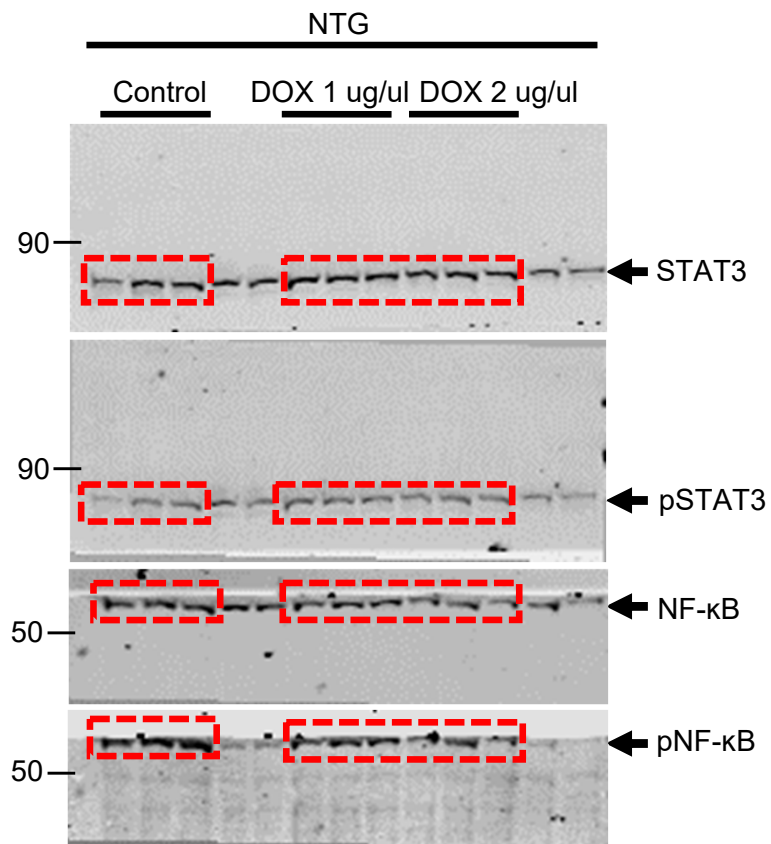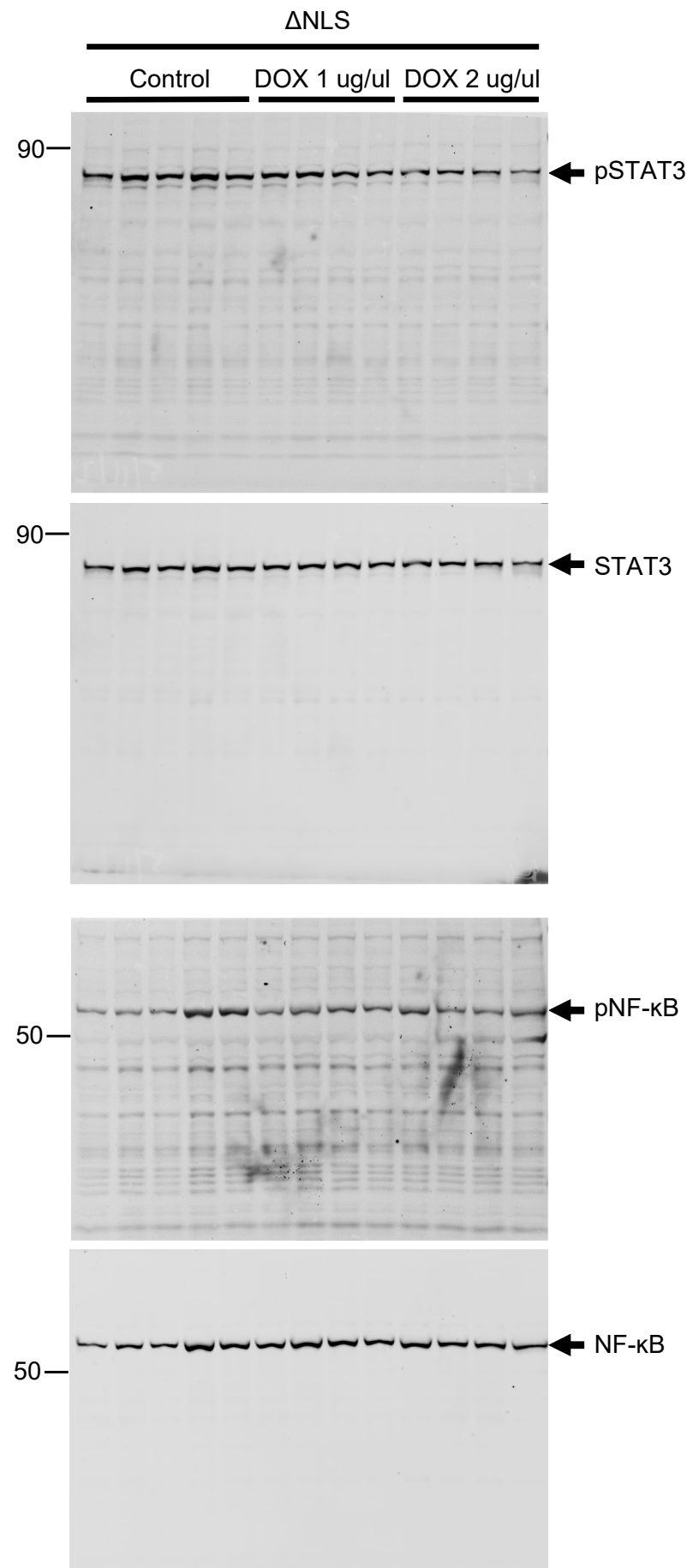

**fig. S1E**

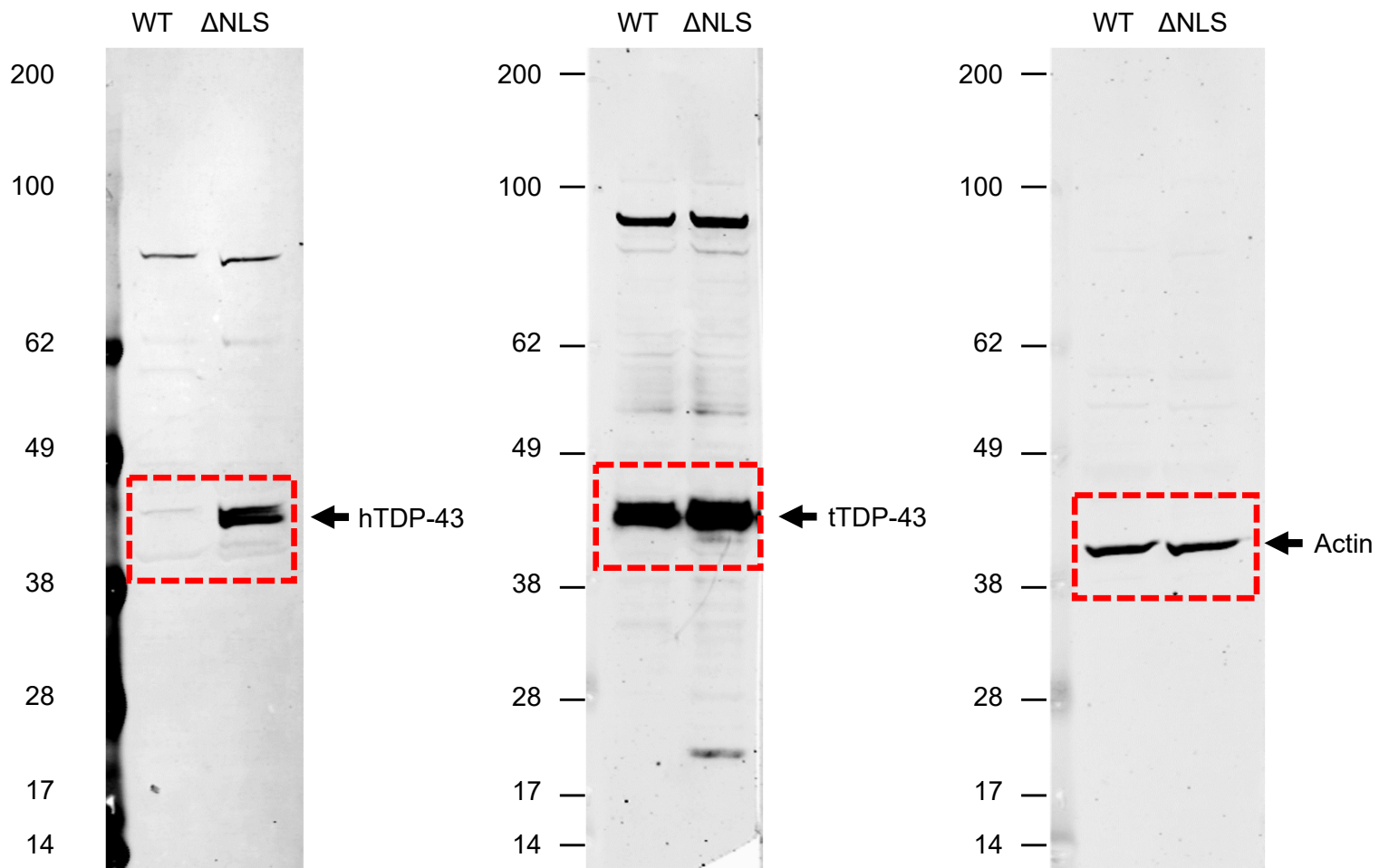

**fig. S7D**

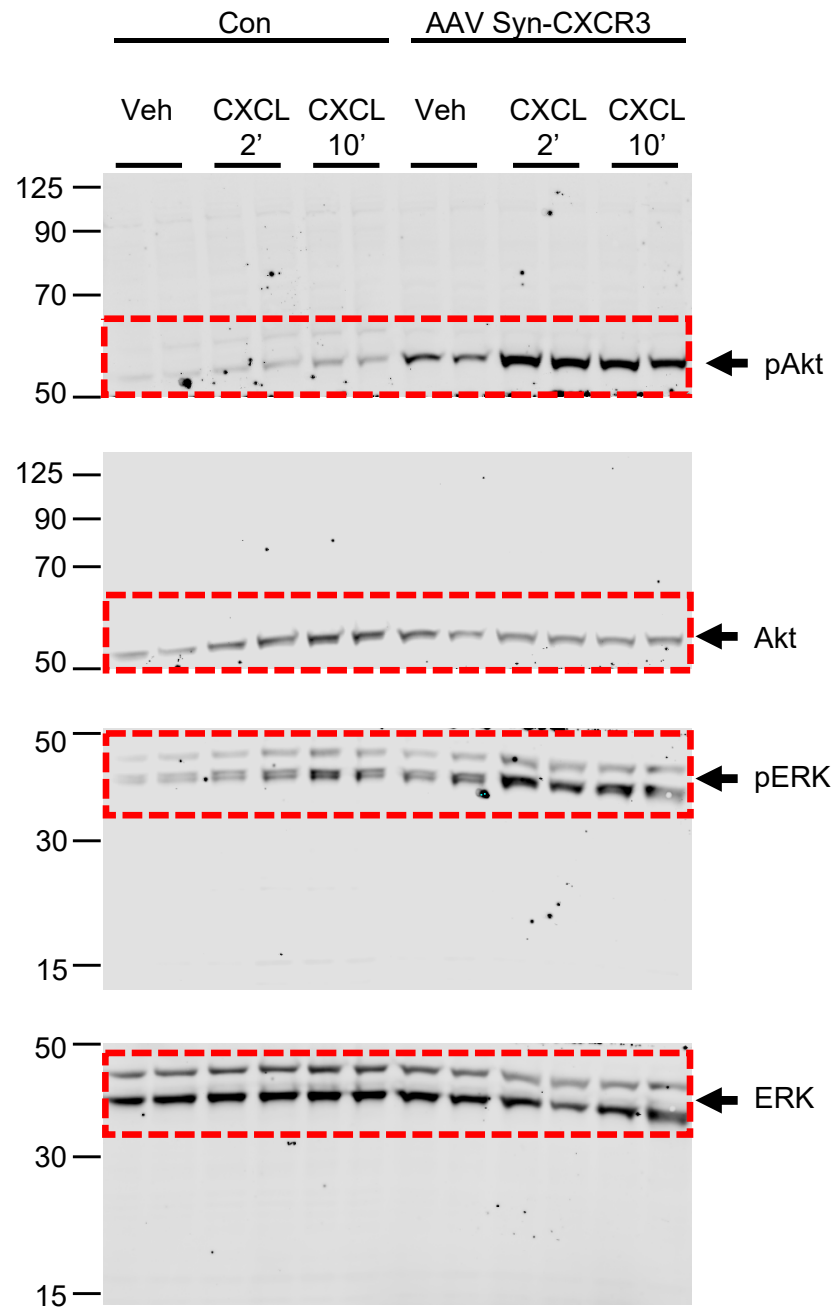

Supplement: Supplementary file 4 — Supplementary Uncropped Western Blot Images [file sciadv.ade1282_supplementary_uncropped_western_blot_images.zip › ade1282_Supplementary Uncropped Western Blot Images.pdf]
